# Genetic Mapping and Characteristics of Genes Specifically or Preferentially Expressed during Fiber Development in Cotton

**DOI:** 10.1371/journal.pone.0054444

**Published:** 2013-01-25

**Authors:** Ximei Li, Daojun Yuan, Jinfa Zhang, Zhongxu Lin, Xianlong Zhang

**Affiliations:** 1 National Key Laboratory of Crop Genetic Improvement (Wuhan), Huazhong Agricultural University, Wuhan, Hubei, China; 2 Department of Plant and Environmental Sciences, New Mexico State University, Las Cruces, New Mexico, United States of America; Wuhan University, China

## Abstract

Cotton fiber is an ideal model to study cell elongation and cell wall construction in plants. During fiber development, some genes and proteins have been reported to be specifically or preferentially expressed. Mapping of them will reveal the genomic distribution of these genes, and will facilitate selection in cotton breeding. Based on previous reports, we designed 331 gene primers and 164 protein primers, and used single-strand conformation polymorphism (SSCP) to map and integrate them into our interspecific BC_1_ linkage map. This resulted in the mapping of 57 loci representing 51 genes or proteins on 22 chromosomes. For those three markers which were tightly linked with quantitative trait loci (QTLs), the QTL functions obtained in this study and gene functions reported in previous reports were consistent. Reverse transcription-polymerase chain reaction (RT-PCR) analysis of 52 polymorphic functional primers showed that 21 gene primers and 17 protein primers had differential expression between Emian22 (*Gossypium hirsutum*) and 3–79 (*G. barbadense*). Both RT-PCR and quantitative real-time PCR (qRT-PCR) analyses of the three markers tightly linked with QTLs were consistent with QTL analysis and field experiments. Gene Ontology (GO) categorization revealed that almost all 51 mapped genes belonged to multiple categories that contribute to fiber development, indicating that fiber development is a complex process regulated by various genes. These 51 genes were all specifically or preferentially expressed during fiber cell elongation and secondary wall biosynthesis. Therefore, these functional gene-related markers would be beneficial for the genetic improvement of cotton fiber length and strength.

## Introduction

Cotton fiber, the seed hair of cotton, is one of the longest single cells in higher plants, and originates from the epidermal cells in the ovular surface. In cultivated species, mature fiber cells are 3.0 to 4.0 cm in length and ∼15 µm in thickness [1]. These mature fibers are almost completely composed of cellulose, which occupies approximately 90% of the dry weight of cotton fibers [2]. It is well known that fiber development is composed of four overlapping stages: fiber cell enlargement and initiation from −3 to 1 day post anthesis (DPA), fiber elongation after anthesis until 25 DPA, secondary cell wall cellulose deposition from 15 to 50 DPA, and fiber cell dehydration and maturation after 45 DPA [3]. This makes the cotton fiber a perfect experimental model to study the mechanism of plant cell elongation, wall development and cellulose biosynthesis [2], [4].

A large number of genes participating in some biological pathways are actively expressed during fiber development. Interestingly, they are differently expressed during different stages. Therefore, identification and analyses of genes preferentially expressed in the different stages will facilitate further understanding of fiber cell elongation and cellulose biosynthesis, and will ultimately result in improved cotton fiber quality and yield.

Thus far, a large number of genes that are specifically or preferentially expressed during fiber development have been identified and isolated. Li et al. [5] found the differential expressions of fifteen *GhACT* cDNAs. In particular, *GhACT1* was predominantly expressed in fiber cells, and played an important role in fiber elongation but not in fiber initiation. It is based on the fact that suppression of *GhACT1* disrupted the actin cytoskeleton and reduced fiber elongation. Another series of genes, 1-Aminocyclopropane-1-CarboxylicAcidOxidase1–3 (*GhACO1*-*GhACO3*), were also expressed at a significantly high level in rapidly elongating fiber cells [6]. Ectopic expression of *GhDET2* in cotton ovule episperm resulted in more fiber initiations and longer fibers [7]. Jiang et al. [8] identified the vital function of *GhSusA1*, a new sucrose synthase gene in fiber strength, boll size and seed weight.

Although cDNA microarray can efficiently analyze cotton fiber development, expression levels of mRNA do not reflect the actual levels of protein. It has been reported that it is necessary to combine transcriptomics with proteomics [9]. Yang et al. [10] have also shown that analysis of proteins was more significant to genome analysis, because proteins existed in the key position between genome and phenotypes. As a result, mapping of markers, which are associated with proteins expressed specifically or preferentially during fiber development in cotton, is important for the genetic improvement of cotton fiber.

Thus far, a large number of differentially expressed proteins have been identified. Using a comparative proteomics approach, Yang et al. [10] found that 235 out of 1800 proteins were differently expressed in elongating fibers. Among these, 120 showed a more than 2-fold change in at least one developmental stage of cotton fiber. More interestingly, 21 of the 235 proteins were expressed specifically at certain stages. Later, 104 differently expressed proteins were identified in cotton ovules 10 DPA using a comparative proteomics approach. And 93 of these accumulated preferentially in the wild-type, while 11 others accumulated in the fuzzless-lintless mutant [11]. In *Li*_1_ fibers, 81 differentially expressed proteins assigned to different functional categories were identified using 2-DE and local EST database-assisted MS/MS analysis. Among these, 54 were down-regulated, and 27 others were up-regulated [12].

However, a high-density molecular map with loci for fiber genes is not available for cotton, primarily due to the lack of fiber gene sequences, and the limited number of simple PCR-based DNA markers available in the public domain [13]. Functional genomics and next generation sequencing (NGS) technologies have enabled the development of markers from genes or coding regions [14]. Currently, some genes related to fiber development have been mapped. For example, *FbL2A*, an important gene for fiber development, was mapped on Chr14 [15]; *GhSAMS* and *GhNLP*, genes related to fiber elongation, were mapped on Chr14 (D2) and Chr19 (D5), respectively [16].

Mapping of the existing genes will reveal their distribution on the linkage map [17], provide a basis for map-based cloning, and contribute to improvement of fiber length and strength using marker-assisted selection (MAS). A high-density linkage map including 2316 loci and enriched by new markers has been constructed in our laboratory [18], [19], [20], [21], and traits related to fiber quality have been explored. In this study, markers were developed from genes specifically or preferentially expressed during cotton fiber development to (i) understand their genomic distribution, (ii) determine their relationship to QTLs involved in fiber quality, and (iii) explore expression differences between *G. hirsutum* and *G. barbadense*.

## Materials and Methods

### Plant materials

*Gossypium hirsutum* cultivar ‘Emian22’ and *G. barbadense* accession ‘3–79’, which are the parents of the BC_1_ mapping population [(Emian22×3–79)×Emian22] [18], [22], were used to detect polymorphisms of the designed functional markers using SSCP. ‘Emian22’ is a high yield cultivar with moderate fiber quality, while ‘3–79’ is the genetic and cytogenetic standard line for *G. barbadense* with super fiber quality. Fiber quality of the parents with four repeats and the BC_1_ population was determined in 2005 according to the methods described by Li et al. [19] (Table 1).

**Table 1:** Fiber quality of Emian22 and 3–79.

	Length(mm)	Uniformity(%)	Micronaire	Elongation(%)	Strength(cN/tex)
**Emian22**	26.80±0.30	82.83±0.46	6.05±0.26	7.08±0.33	24.90±0.32
**3–79**	33.86±0.76**	85.90±1.43**	4.41±0.19**	4.68±0.50**	37.60±1.68**

** *P*≤0.01.

### Primer design

The assembled cotton gene\EST sequences were downloaded from GenBank (http://www.ncbi.nlm.nih.gov/genbank) using the accession numbers from previous reports (Table S1). tblastn at NCBI was used to obtain the nucleotide sequences of proteins specifically or preferentially expressed during fiber development. Sequence-specific primers were designed using Primer-BLAST (http://blast.ncbi.nlm.nih.gov/Blast.cgi) with the following criteria: length of primer ranging from 18 to 30 bp, primer Tm ranging from 57 to 63°C, difference of Tm between the two primers within a pair less than 3°C, predicted PCR products ranging from 100 to 400 bp, and GC content ranging from 40 to 60%. Primers designed from genes were given the gene names (Table S1), and those designed from proteins were named as FPG+primer number (Table S2). If more than one marker was developed from the same sequence, then numbers such as 1, 2, etc. were used as suffix. All primers including 331 gene primers and 164 protein primers were synthesized by sunbiotechnology (Beijing, China).

### SSCP analysis

PCR amplification was carried out according to the methods described by Lin et al. [23]. All markers were subjected to polymorphism detection using SSCP analysis described by Li et al. [19]. For the remnant monomorphic markers, improved SSCP analysis was applied at a constant watt of 8W for about 6 h at 4°C. Subsequently, genotyping of the whole population using polymorphic primers was carried out on the corresponding condition. All DNA fragments were detected with silver staining.

### Map construction and QTL analysis

The polymorphic loci were integrated into the interspecific BC_1_ linkage map [18], [19], [20], [21], and QTL mapping was performed based on newly improved linkage map. Both map construction and QTL mapping were carried out according to the methods described by Li et al. [19].

### RT-PCR and qRT-PCR analyses

RNAs were extracted from cotton fibers at different stages in development (0, 5, 10, 15, 20 and 25 DPA). First strand cDNA synthesize, RT-PCR and qRT-PCR analyses were performed according to the methods described by Munis et al. [24] with minor modifications. Ubiquitin (GenBank: AY375335) was used as an internal control, and a gene specific primer pair (forward 5′ GAAGGCATTCCACCTGACCAAC 3′, reverse 5′ CTTGACCTTCTTCTTCTTGTGCTTG 3′) was used to amplify ubiquitin. In addition, RT-PCR experiments had two repetitions, and qRT-PCR experiments had three repetitions.

### GO analysis

Functional annotation of nucleotide sequences containing mapped markers was performed using Blast2GO [25], [26] with default parameters. Analyses were conducted as follows: (1) blastx [27] analysis was carried out with E-value of 1.0 E^−3^ against nr protein database in GenBank, and the top twenty blast hits were retained. (2) GO-mapping, annotation analysis, InterPro Scan and InteProScan GOs was performed one after another. (3) GO-Slim (http://www.geneontology.org/GO.slims.shtml) was then carried out to acquire specific GO terms. (4) Statistical analysis was done lastly to classify sequences into one of the three categories, cell component, molecular function, and biology process on levels 1, 2 and 3.

## Results

### Development and polymorphism of functional markers

To map genes and proteins specifically or preferentially expressed during fiber development in cotton, target sequences from previous reports were used to design primers. In total, 331 gene primers and 164 protein primers were designed using Primer-BLAST (http://blast.ncbi.nlm.nih.gov/Blast.cgi). Of these markers, 199 (60.12%) gene primers and 114 (69.51%) protein primers amplified target products. After genotyping using two different SSCP methods, 33 gene primers (9.97%) and 19 protein primers (11.59%) were found to be polymorphic, and finally 37 polymorphic loci for genes and 21 for proteins were generated. Details of the 52 polymorphic functional markers are listed in Table 2.

**Table 2:** Details of polymorphic markers.

Marker name	Chromosome	Subgenome	GenBank	Kind of SSCP analysis	Results of RT-PCR
*Gh14-3-3a*-ss	25	D	EU189220	1	×
*GhADF6*-ds	15	D	DQ402083	1	★
*GhAPY1*-ss	13	A	GU385147	2	▴
*GhAPY2*-ss	11	A	GU385148	2	▴
*GhBCP4*-ss	21	D	GU451702	2	★
*GhbHLH1*-ds	8	A	FJ358540	1	▴
*GhCAD6*-ss	26	D	EU281305	1	★
*GhCESA3*-ss	8	A	AF150630	1	★
*GhDET3*-ds	5	A	DQ838492	1	★
*GhEXPA4-2*-ds	9	A	AF512542	1	★
*GhEXPA6-3*-ds	3	A	AF512544	1	×
*GhFb-B6-2*-ds	6	A	U13760	1	▴
*GhFLA10*-ds	13	A	EF672636	1	▴
*GhFLA14*-ss	23	D	EF672640	1	★
*GhFLA14*-dsa	9	A	EF672640	1	★
*GhFLA14*-dsb	9	A	EF672640	1	★
*GhFLA17*-ss	25	D	EF672643	2	×
*GhFLA19*-ds	16	D	EF672645	1	×
*GhGal1*-ds	15	D	AY438035.1	2	★
*GhGlcAT1*-ss	9	A	AY346330.1	2	▴
*GhGLP1*-ss	20	D	AF116537	1	√
*GhGS*-ds	16	D	EU223825	1	×
*GhKCR3*-ss	8	A	AY902468	1	★
*GhLipase*-ssa	13	A	EU273289	1	×
*GhLipase*-dsb	18	D	EU273289	1	×
*GhMYB102a*-ds	21	D	AJ459117	1	√
*GhPEAMT*-ds	3	A	EF688569	1	★
*GhPEPC2*-ds	15	D	EU032328	1	▴
*GhPIP1-2*-ssa	24	D	DR176739	1	★
*GhPIP1-2*-dsb	24	D	DR176739	1	★
*GhSLR1b*-ss	14	D	AY208992	1	×
*GhSUT-2*-ss	10	A	AY375329	2	▴
*GhTF1*-ss	9	A	EF651783	1	×
*GhTUB7*-ss	3	A	AY345609	2	★
*GhUGT2*-ss	16	D	EF408256	2	×
*LMWHSP1*-ds	4	A	EU223832	2	×
*LMWHSP2*-ds	24	D	EU223833	1	★
FPG012-ss	16	D	EU223825	1	★
FPG013-ss	26	D	HM370495	1	√
FPG019-ss	19	D	U73746	2	▴
FPG028-ss	16	D	AY429443	2	▴
FPG035-ss	11	A	AY072824	1	▴
FPG061-ssa	18	D	DW231397	2	▴
FPG061-ssb	13	A	DW231397	2	▴
FPG065-ss	19	D	CO074226	1	▴
FPG075-ss	26	D	DT049468	2	★
FPG088-ss	19	D	EV485102	2	★
FPG090-ss	10	A	DT052785	1	★
FPG100-ss	22	D	DW234018	1	▴
FPG105-ss	13	A	ES815260	1	▴
FPG123-ss	25	D	FJ415196	1	★
FPG132-ss	8	A	FJ415189	2	★
FPG136-ss	17	D	EU376003	2	★
FPG137-ss	26	D	AF521252	1	√
FPG139-ss	22	D	FJ415176	1	▴
FPG151-ss	none	neither	FJ415166	2	▴
FPG157-ssa	22	D	GU295063	2	★
FPG157-ssb	5	A	GU295063	2	★

1: Traditional SSCP analysis method described by Li et al. (2012).

2: Improved SSCP analysis method described in this study.

×: No or weak expression in both Emian22 and 3–79.

√: Absence of differential expression between Emian22 and 3–79.

▴: Minor difference in expression between Emian22 and 3–79.

★: Obvious difference in expression between Emian22 and 3–79.

### Distribution of functional markers on the interspecific BC_1_ genetic linkage map

**Figure 1:**
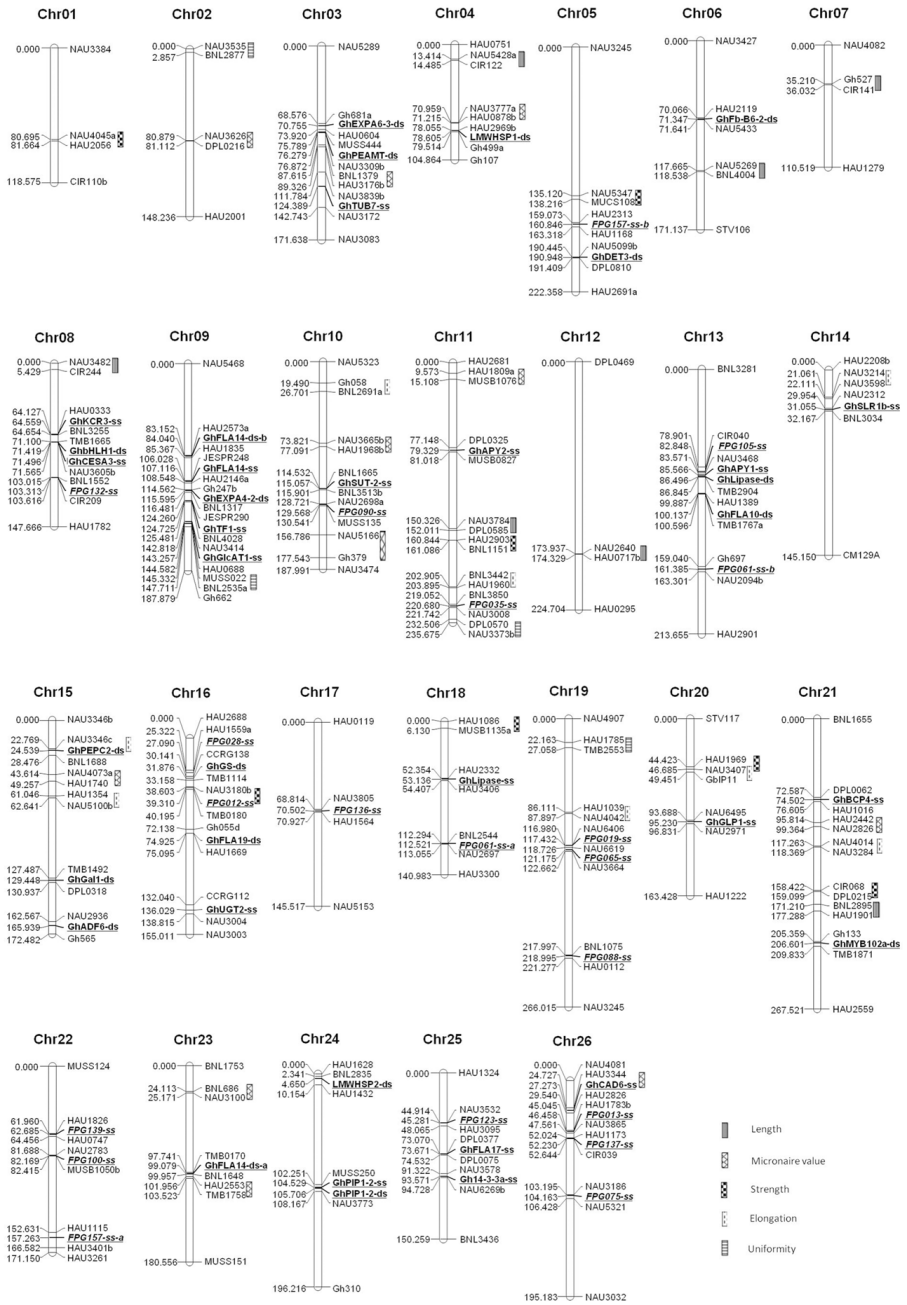
Locations of polymorphic markers and fiber-related QTLs on the BC_1_ genetic linkage map. Gene markers are underlined and in bold. Protein markers are underlined, italicized and in bold.

Markers that detected clear and scorable polymorphisms between Emian22 and 3–79 were selected to survey the BC_1_ population. After linkage analysis, 57 loci representing 51 genes or proteins except FPG151 were added to the existing interspecific BC_1_ genetic linkage map. Twenty-five loci were mapped on 9 chromosomes of the At subgenome, and 32 loci on 13 chromosomes of the Dt subgenome. Generally, they were distributed randomly on 22 chromosomes: 5 loci were mapped on Chr09, Chr13 and Chr16, respectively; 4 loci on Chr08 and Chr26, respectively; 3 loci on Chr03, Chr15, Chr19, Chr22, Chr24 and Chr25, respectively; 2 loci on Chr05, Chr10, Chr11, Chr18 and Chr21, respectively; 1 loci on Chr04, Chr06, Chr14, Chr17, Chr20 and Chr23, respectively (Fig. 1, Table 2).

### Mapping of fiber related QTLs

QTL scanning was performed based on the latest linkage map improved by polymorphic functional markers from this study. Among all the QTLs, three were tightly linked with functional markers developed in this study: *GhPEPC2*-ds on Chr15 was tightly linked with *qFEchr15.1* which explained 7.35% of the total phenotypic variance explained (PVE); FPG012-ss on Chr16 was tightly linked with *qFSchr16* (4.48% PVE); and *GhCAD6*-ss on Chr26 was tightly linked with *qMVchr26* (8.07% PVE) (Fig. 1, Table 3).

**Table 3:** Details of the three QTLs tightly linked with functional markers.

QTL Name	Position	Left Marker	Right Marker	LOD	PVE (%)	Additive
*qMVchr26*	25.00	HAU3344	*GhCAD6*-ss	5.59	8.07	0.31
*qFSchr16*	39.00	NAU3180b	FPG012-ss	2.55	4.48	1.06
*qFEchr15.1*	25.00	*GhPEPC2*-ds	BNL1688	4.18	7.35	−0.30

### Expression difference between *G. hirsutum* and *G. barbadense*

**Figure 2:**
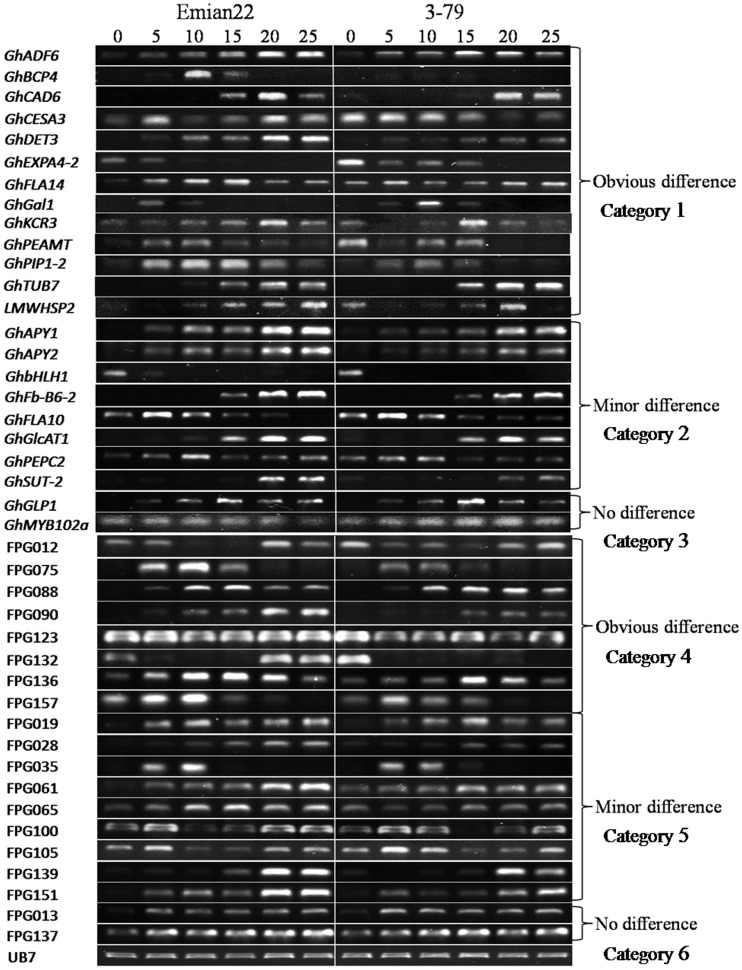
RT-PCR analysis of 42 polymorphic and productive primers. Numbers on the top represent 0DPA, 5DPA, 10DPA, 15DPA, 20DPA and 25DPA, respectively. Primers, which have either differential expression tendencies or obvious differential expression levels between Emian22 and 3–79, were classified into the obvious difference category. Primers with minor difference in expression levels between Emian22 and 3–79 were classified into the minor difference category. Primers with neither differential expression tendencies nor differential expression levels between Emian22 and 3–79 were classified into the no difference category. Gene primers and protein primers are labeled on the left.

RT-PCR analysis of 33 polymorphic gene primers showed that between Emian22 and 3–79, 13 showed obvious differential expression, 8 showed minor difference, and 2 showed no difference, while 10 (30.30%) had no or weak expression in both Emian22 and 3–79 (Table 2). Among the 19 polymorphic protein primers, 8 showed obvious differential expression between Emian22 and 3–79, and 9 showed minor difference. The remaining 2 primers showed no difference between Emian22 and 3–79 (Table 2). Expression levels of 23 gene primers and 19 protein primers at every stage of fiber development are presented in Fig. 2. To further confirm the results of RT-PCR, randomly chosen genes belonging to different categories were used in the qRT-PCR analysis (Fig. S1). Consistent results were observed in both RT-PCR and qRT-PCR analyses.

### Functional categorization of sequences using GO analysis

GO analysis assigned 125 functions to 51 sequences, with 42 each in cell component and biology process categories, and 41 in the molecular function (Fig. S2). On level 2, 4 sub-categories were present in cell component, 6 in molecular function, and 12 in biology process (Fig. S3). On level 3, 4 sub-categories were observed in cell component, 9 in molecular function, and 21 in biology process (Fig. S4).

## Discussion

### Low polymorphism of functional markers

To detect polymorphisms and to genotype the mapping population, SSCP was applied in this study primarily because it is inexpensive, easy to handle, does not require special devices, and was presumed to facilitate genome wide mapping of cotton [19], [28]. We found that primer polymorphism of the functional markers (10.51%) was relatively low, which is consistent with the fact that markers derived from coding sequences have lower polymorphism because of their more highly conserved nature than non-coding sequences [29]. Although such low polymorphism could be partly due to the shortcoming of SSCP, these results also indicate the highly conserved nature of the analyzed genes between *G. hirsutum* and *G. barbadense*. Noteworthily, the percentage of amplified protein primers (69.51%) was slightly higher than gene primers (60.12%), suggesting that differently translated proteins are more reliable than transcripts. Theoretically, interspecific polymorphism of protein primers is higher than gene primers because the relevance between proteins and genotypes is higher than that between genes and genotypes [10]. Therefore, isolation of differentially expressed proteins would be more effective in the identification of functional genes involved in the fiber development of cotton.

### Even distribution of functional markers on the interspecific linkage map

Recently mapped fiber EST-derived simple sequence repeats (SSRs) showed the equal importance of At and Dt subgenomes in fiber development [30]. The result of our study, which showed an approximately equal distribution of functional markers between the two subgenomes, is consistent with the previous report. However, we identified a few more loci on Dt than At subgenome. A previous RFLP-QTL mapping study showed that most QTLs related to fiber quality and yield were located on Dt subgenome of cultivated tetraploid cotton [31]. In addition, it has been reported that Chr05, Chr10, Chr14 and Chr15 were rich in genes involved in fiber development [1]. However, fiber-related genes in this study were distributed randomly throughout the cotton genome. This inconsistency could be attributed to the low level of DNA marker polymorphisms and the limited number of candidate fiber gene markers mapped [32] with only 57 loci of 51 functional markers from 495 mapped on chromosomes. This indicated that cotton fiber development was regulated by the whole genome.

### Functional markers for fiber development associated with fiber-related QTLs

Primer FPG012 tightly linked with *qFSchr16* was designed from sequence of glutamine synthetase (GS), while *GhGS* was related to fiber strength [33]. Together they enhanced the reliability of *qFSchr16* drastically. Cotton fiber elongation requires high activity of PEPC that ultimately influences fiber length, presumably through the expression of *GhPEPC1* and *GhPEPC2* [34]. However, *GhPEPC2* mapped on Chr15 in this study was tightly linked with *qFEchr15.1* rather than fiber length related QTLs. We observed a slightly discrepancy between QTL function and gene function. Previous reports have shown that genes preferentially expressed during secondary cell wall cellulose deposition have relevance with micronaire [35]. However the thickened secondary walls of mature cotton fibers may not have pure cellulose but could be mixed with phenolics [36]. While phenolics protects cellulose fibers in the plant cell walls [37], their deposition may decrease the plasticity of expanding cell walls and influence the cessation of growth during cell maturation [38]. Because *GhCAD6* mapped on Chr26 in this study was reported to be involved in the biosynthesis of the phenylpropanoid unit and cell wall phenolics [36], this gene could have some relevance to micronaire. The linkage of *GhCAD6* to *qMVchr26* is evidence to the speculative relationship between them. Together these three genes could play important roles in cotton fiber development.

### Functional markers for fiber development differentially expressed between *G. hirsutum* and *G. barbadense*

The efficiency of protein primers (100.00%) determined by RT-PCR was higher than gene primers (69.70%), which further confirmed that isolation of differentially expressed proteins were more effective than genes. In addition, RT-PCR analysis revealed the differential expression of some genes between *G. hirsutum* and *G. barbadense* (Fig. 2), which was also confirmed by qRT-PCR (Fig. S1). These expression patterns provide some guidance to improve fiber quality of *G. hirsutum*, which could be accomplished by transforming those genes preferentially expressed in *G. barbadense* and controlling fiber quality obviously.

Reliability of the three QTLs tightly linked with functional markers developed in this study was shown by RT-PCR. Expression of primer FPG012, tightly linked with *qFSchr16*, was observed in more stages in 3–79 than Emian22 (Fig. 2), while fiber strength of 3–79 is drastically higher than Emian22 (Table 1). It has been reported that fiber strength is mainly relative to the secondary cell wall cellulose deposition, which occurs from 15 to 50 DPA [3]. The results of RT-PCR showed a more robust expression of primer FPG012 in later stages in 3–79 than Emian22, because the expression peak appeared at 20 DPA in Emian22, while it might appear at later stages in 3–79 (Fig. 2). A similar result was also observed in the qRT-PCR analysis of FPG012 (Fig. S1e).

*GhPEPC2* has been reported to be highly expressed during the rapid elongation phase but weakly expressed at the slow-to-terminal elongation period [34]. Both results of RT-PCR and qRT-PCR in this study showed that both Emian22 and 3–79 had similar expression tendencies with previous report (Fig. 2, Fig. S1d). However, minor differences existed between them with expression levels in Emian22 higher than in 3–79. Considering that fiber elongation of Emian22 is higher than 3–79 (Table 1), this result gave some evidence to *qFEchr15.1* tightly linked with *GhPEPC2*.

*GhCAD6*, tightly linked with *qMVchr26* in this study, was expressed from 15 to 25 DPA in Emian22 while in 3–79 the expression started at 20 DPA (Fig. 2). Similar results were obtained from qRT-PCR analysis of *GhCAD6* (Fig. S1a). Fiber quality of the two parents showed that micronaire of Emian22 was higher than 3–79 (Table 1). Results between RT-PCR analysis and QTL analysis were mutually verified.

### Complexity of cotton fiber development

Based on the GO analysis, 51 genes were grouped into three functional categories (Fig. S2). There was no obvious difference of number among them. Most genes belonged to two or three categories, which illustrated the involvement of genes in multiple processes and the complexity of fiber development. On level 2, no distinct differences were observed, too. On level 3, the largest proportion in the molecular function category was the hydrolase activity (24.39%). Because fiber development is closely related to hydrolase activity [39], these genes may be important for the genetic improvement of fiber. Biosynthetic process that had the largest proportion (33.33%) in the biology process category was also closely relevant to fiber development [40]. Because fiber itself is a single cell, it is plausible that fiber development-related genes were largely involved in cell part, which comprised nearly all of cell component category.

## Supporting Information

Figure S1 **qRT-PCR analysis of nine primers randomly chosen from different categories.** Expression levels of Emian22 (black column) and 3–79 (grey column) are shown. “*” represents *P*≤0.05, and “**” represents *P*≤0.01. Primers *GhCAD6* (a) and *GhPIP1-2* (b) were chosen from category 1; *GhAPY1* (c) and *GhPEPC2* (d) from category 2; FPG012 (e) and FPG157 (f) from category 4; FPG061 (g) and FPG065 (h) from category 5. Primer FPG013 (i) was chosen as the representative of categories 3 and 6, which showed no difference in expression between Emian22 and 3–79.
(TIF)

Figure S2 **Functional categorization of sequences on level 1.** Distribution of the 51 functional sequences among the three categories (cell component, molecular function and biology process categories).
(TIF)

Figure S3 **Functional categorization of sequences on level 2.** Four sub-categories in cell component category (a), 6 in molecular function category (b), and 12 in biology process category (c).
(TIF)

Figure S4 **Functional categorization of sequences on level 3.** Four sub-categories in cell component category (a), 9 in molecular function category (b) and 21 in biology process category (c).
(TIF)

Table S1 **Details of the 331 gene primers.** Gene primer names, forward and reverse primer sequences, and references of the nucleotide sequences used to design gene primers are all listed.
(XLS)

Table S2 **Details of the 164 protein primers.** Protein primer names, forward and reverse primer sequences, and references of the amino acid sequences consulted to design protein primers are all listed.
(XLS)
