# Supplementary figures and images for: Genetic Mapping and Characteristics of Genes Specifically or Preferentially Expressed during Fiber Development in Cotton
Source: PLoS One. 2013 Jan 25;8(1):e54444. doi: 10.1371/journal.pone.0054444 (PMC3555819; doi:10.1371/journal.pone.0054444)

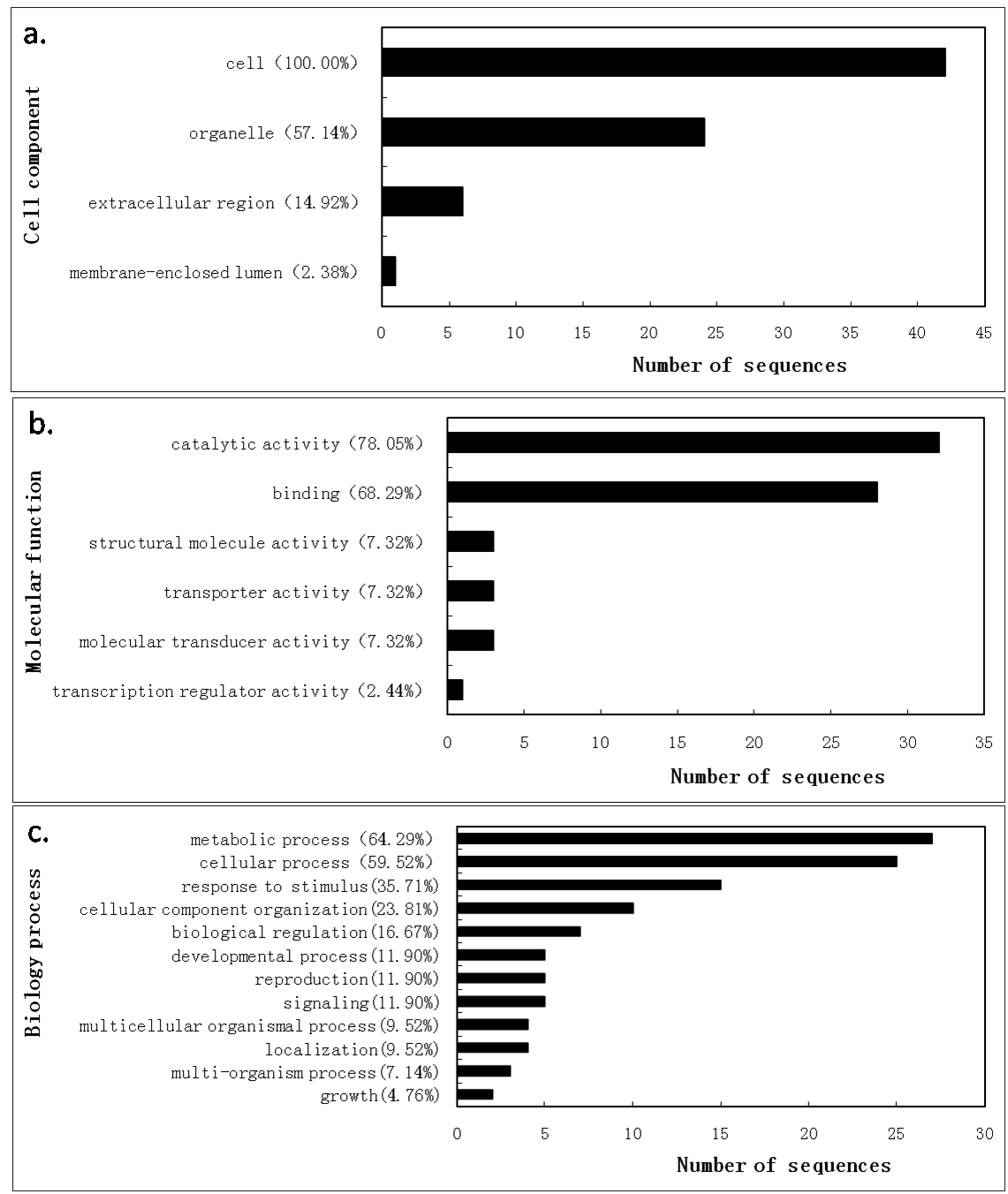

Supplement: Figure S3 — Functional categorization of sequences on level 2. Four sub-categories in cell component category (a), 6 in molecular function category (b), and 12 in biology process category (c). (TIF) [file pone.0054444.s003.tif]

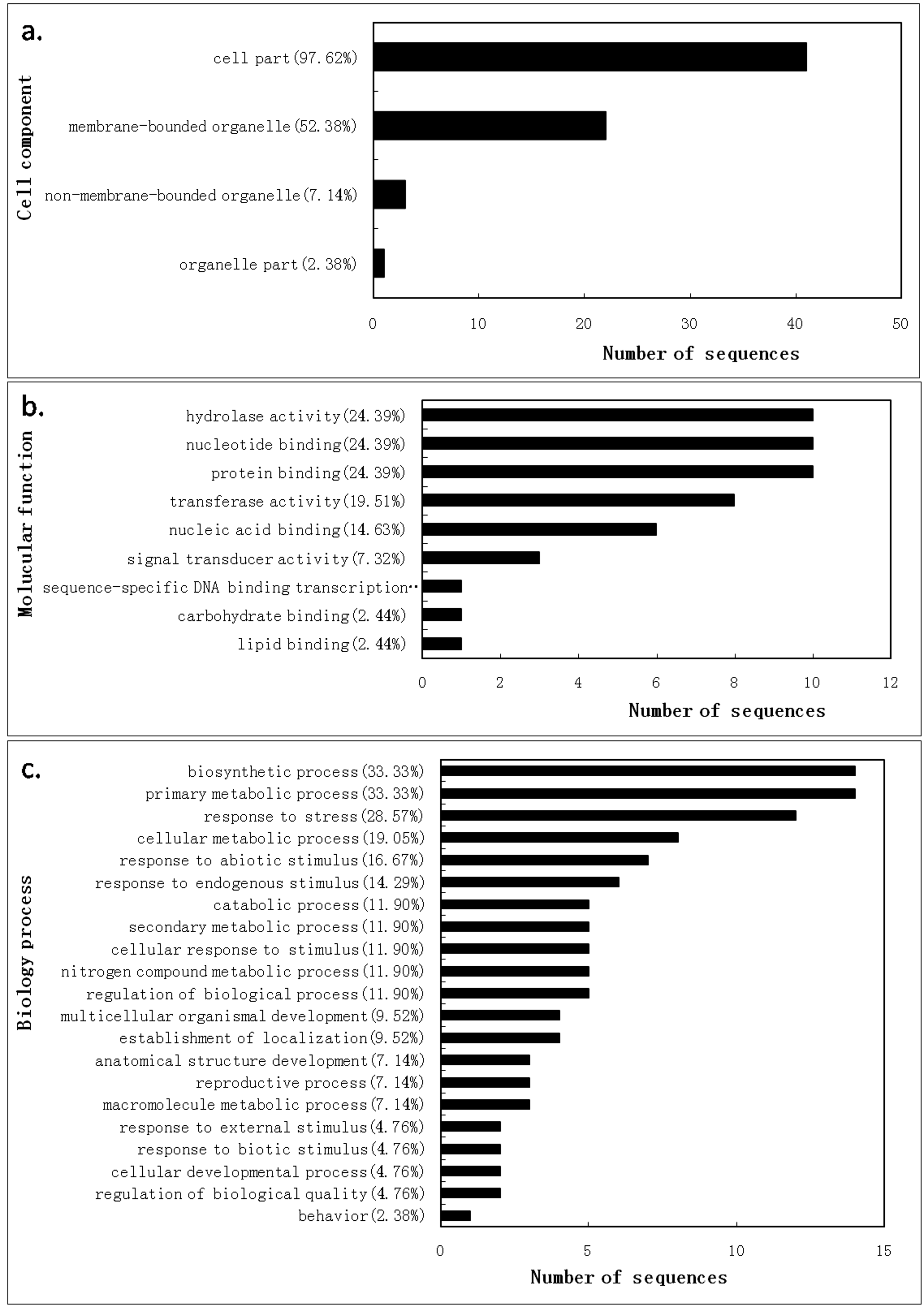

Supplement: Figure S4 — Functional categorization of sequences on level 3. Four sub-categories in cell component category (a), 9 in molecular function category (b) and 21 in biology process category (c). (TIF) [file pone.0054444.s004.tif]
